# Comparison study of differential abundance testing methods using two large Parkinson disease gut microbiome datasets derived from 16S amplicon sequencing

**DOI:** 10.1186/s12859-021-04193-6

**Published:** 2021-05-25

**Authors:** Zachary D. Wallen

**Affiliations:** Department of Neurology, University of Alabama At Birmingham, Birmingham, AL 35294 USA

**Keywords:** Differential abundance, Microbiome, 16S, Parkinson disease

## Abstract

**Background:**

Testing for differential abundance of microbes in disease is a common practice in microbiome studies. Numerous differential abundance (DA) testing methods exist and range from traditional statistical tests to methods designed for microbiome data. Comparison studies of DA testing methods have been performed, but none performed on microbiome datasets collected for the study of real, complex disease. Due to this, DA testing was performed here using various DA methods in two large, uniformly collected gut microbiome datasets on Parkinson disease (PD), and their results compared.

**Results:**

Overall, 78–92% of taxa tested were detected as differentially abundant by at least one method, while 5–22% were called differentially abundant by the majority of methods (depending on dataset and filtering of taxonomic data prior to testing). Concordances between method results ranged from 1 to 100%. Average concordance for datasets 1 and 2 were 24% and 28% respectively, and 27% for replicated DA signatures. Concordances increased when removing rarer taxa before testing, increasing average concordances by 2–32%. Certain methods consistently resulted in higher concordances (e.g. ANCOM-BC, LEfSe), while others consistently resulted in lower (e.g. edgeR, fitZIG). Hierarchical clustering revealed three groups of DA signatures that were (1) replicated by the majority of methods on average and included taxa previously associated with PD, (2) replicated by a subset of methods and included taxa largely enriched in PD, and (3) replicated by few to one method(s).

**Conclusions:**

Differential abundance tests yielded varied concordances, and amounts of detected DA signatures. Some methods were more concordant than others on both filtered and unfiltered data, therefore, if consistency with other study methodology is a key goal, one might choose among these methods. Even still, using one method on one dataset may find true associations, but may also detect false positives. To help lower false positives, one might analyze data with two or more DA methods to gauge concordance, and use a built-in replication dataset. This study will hopefully serve to complement previously reported DA method comparison studies by implementing and coalescing a large number of both previously and yet to be compared methods on two real gut microbiome datasets.

**Supplementary Information:**

The online version contains supplementary material available at 10.1186/s12859-021-04193-6.

## Background

Microbiome research has gained immense traction in recent years driven primarily by technological advances in sequencing and exponential increase in computational resources and tools. The availability of these new tools and technologies have solidified a place for microbiome research in many fields including the biomedical research community where a large portion of the research effort is targeted at the gut microbiome [1]. A number of diseases have been associated with alterations of individual microorganisms in the gut [1], and these associations are usually made through a statistical analysis commonly referred to differential abundance (DA) testing [2]. Differential abundance testing involves the use of statistical testing to determine if the relative abundances of certain microorganisms are significantly different between defined groups [3]. Numerous DA testing methods exist and include classical statistical tests (e.g. Kruskal–Wallis rank-sum test), methods originally developed to detect differential expression of gene transcripts in RNA-Seq data and adapted for microbiome analysis (e.g. DESeq2, edgeR), methods specifically designed for detecting differentially abundant microorganisms in microbiome data (e.g. ANCOM, metagenomeSeq), and methods designed to detect differentially abundant features (whether it be microorganisms or gene transcripts) in compositional data (e.g. ALDEx2). Differences in choice of DA method can contribute to inter-study variation in results, even between studies of the same disease, as most, if not all, methods will respond differently to microbiome data due to differences in their underlying characteristics. Multiple studies have previously assessed and compared the performance of popularly used DA testing methods, measuring their false positive rates (FPR), false discovery rates (FDR), sensitivities, and/or specificities using simulated data [2–5], with only one of these studies testing different methods on real data [4]. An example of how DA testing methods compare to one another when performed on real, complex disease gut microbiome datasets is still lacking in the literature. Also, not all methods included in previous comparison studies have been compared side by side as each study compared few to several methods at a time with slight differences in what methods were included in their assessments and comparisons.

Due to the lack of literature on how different DA methods behave and compare to one another when performed on real, complex disease gut microbiome datasets, DA testing was performed here using a variety of methods on two, large Parkinson disease (PD)—gut microbiome datasets in order to compare their results. Commonly used DA testing methodologies were found in the literature, and used to test for differentially abundant microbial genera (referred to as “DA signatures”) between PD and neurologically healthy controls in both datasets. DA testing was performed once for all genera that were detected in each dataset (referred to as “unfiltered” data), and again for only genera that were detected in at least 10% of samples in each dataset (referred to as “filtered” data) in order to assess effect of taxonomic data filtering on results. Once DA testing was complete, results were compared within and across datasets. Concordances between methods varied, although a subset of methods consistently resulted in higher concordances on average, especially with one another. These methods also detected DA signatures across datasets that included more prevalent genera and seemed more robust to inter-methodological variation. Another subset of methods consistently produced the least concordant results on average with other methods, but some detected DA signatures in both datasets involving a subset of rarer genera, all (but one) with higher relative abundances in PD. Almost all concordances for individual methods improved when filtering out rarer genera before testing. More concordant methods tended to detect a smaller proportion of genera as differentially abundant compared to lower concordant methods, but this difference was attenuated when rarer genera were removed before analysis. The vast majority of genera tested (78–92%) were detected as differentially abundant by at least one method in each dataset, while 5–22% were called differentially abundant by the majority of methods (depending on dataset and filtering of taxonomic data prior to testing). Fewer signals were actually replicated in both datasets by at least one method (49–68%), and even fewer were replicated by the majority (1–11%) or all (~ 1%) of the methods. Hierarchical clustering revealed three groups of DA signatures that were (1) replicated by the majority of methods on average and included genera previously associated with PD, (2) replicated by a subset of methods and included genera largely enriched in PD, and (3) replicated by few to one method(s). Input data and source code used to perform analyses and create figures and tables can be publicly accessed at the following GitHub repository: https://github.com/zwallen/Wallen_DAMethodCompare_2021.

## Results

### Method characteristics

Methods included in the present study span the fields of traditional statistics, RNA-Seq analysis, and microbiome analysis, and have varying underlying characteristics. A summary of method characteristics and parameters chosen that differed from default for DA methods included in this study can be found in Additional files 2 and 3: Tables S1 and S2 respectively. The DA methods compared in this study included ANCOM [6], ANCOM with bias correction (ANCOM-BC) [7], ALDEx2 (using t-test or Wilcoxon tests) [8], baySeq [9], DESeq2 nbinomWald test [10], edgeR exactTest using relative log expression (RLE) or trimmed mean of M-values (TMM) [11], generalized linear model (GLM), Kruskal–Wallis rank-sum test [12], LEfSe [13], limma-voom [14], metagenomeSeq’s fitFeatureModel and fitZIG [15, 16], negative binomial GLM with or without zero-inflation (GLM NBZI), SAMseq [17], and Welch’s t-test [18]. The majority of methods utilized parametric statistical tests (assumes the data has some form of underlying distribution). Of these, the most commonly assumed data distribution was the negative binomial distribution (DESeq2, baySeq, edgeR RLE, edgeR TMM, GLM NBZI). No data transformations were performed for negative binomial methods, or metagenomeSeq methods, to try and bring the data to normality as non-normality of data is taken into account in their statistical models. The remaining parametric methods (ALDEx2 t-test, t-test, limma-voom, GLM, ANCOM-BC) all used statistical tests that assumed a Gaussian distribution of the data, therefore, transformations were needed before analysis, which here included a log transform of some kind. Five methods (ALDEx2 Wilcoxon, ANCOM, Kruskal–Wallis, SAMseq, LEfSe) were considered non-parametric (assumes no underlying distribution of data) as they used statistical tests that transformed data to ranks. Methods also differed in what techniques were used to account for varying sequence depth between samples. Four of the five negative binomial methods (DESeq2, baySeq, edgeR RLE, edgeR TMM) calculated scaling factors for each sample to account for uneven sequence count. Cumulative sum scaling (CSS) was used for both metagenomeSeq methods. Total sum scaling (TSS; also referred to as relative abundance) was performed for LEfSe, and was one of three strategies used for methods that did not have a built-in normalization function (t-test, GLM, Kruskal–Wallis) as this strategy is commonly used in the literature. Log-ratio based transformations were used for ALDEx2 methods, ANCOM, and, in addition to TSS, were applied to methods without a built-in normalization function. Log-ratio transformations (including centered log-ratio (CLR) [19] and robust CLR with matrix completion (rCLR) [20] used with Kruskal–Wallis, GLM, and t-test) are compositionally aware methods that, in addition to normalizing for inter-sample variation in total sequence count, takes the compositionality of the data into account. While not directly implementing a log-ratio transformation, ANCOM-BC’s bias correction (a sample specific offset term that is introduced into a linear regression and accounts for sampling fraction) in conjunction with performing linear regression in log scale serves the same purpose as a log-ratio transformation. The remaining 3 methods (limma-voom, GLM NBZI, SAMseq) did not share normalization strategies with any other methods. To account for varying sequencing depth, limma-voom utilized a log2-counts per million (CPM) transformation, the log of the per sample sequencing depth was used as an offset variable in the model for GLM NBZI, and SAMseq utilized its own unique transformation which included an Anscombe transformation followed by dividing by square root of the sequencing depth. The majority of methods have been previously included in some form of method comparison study [2–5], and all seemed to keep FPR and/or FDR under 0.2 when performed on simulated data except for fitZIG, edgeR, and SAMseq, which were shown to reach upwards of 0.9 (Additional file 2: Table S1).

### Comparison of method results across datasets

Datasets differed in size and had significant heterogeneity in microbiome composition [21], therefore, method calls were compared across datasets to see if there were any significant dataset differences in the number, or proportion, of DA signatures being detected by DA methods. Within each dataset, 78–92% of genera were detected as differentially abundant by at least one method, 5–22% of genera were detected as differentially abundant by at least half of the methods, and only two genera (*Agathobacter* and *Roseburia*) were detected by all methods, and only when performed on filtered taxonomic data (Additional files 4, 5, 6, 7: Tables S3–S6). No significant difference was found between datasets for the average number of methods that detected a particular DA signature when analyzing filtered data [*t*_(235)_ = − 0.4, *P* = 0.7; Additional file 1], but a small, yet significant, difference was found between datasets when analyzing unfiltered data [*t*_(999)_ = − 5.1, *P* = 3E−7; Additional file 1]. When comparing results across datasets for replication of DA signatures, 49–68% of genera tested and in common between datasets were detected as differentially abundant by at least one method in both datasets, while 1–11% were detected by at least half of the methods in both datasets (Additional files 4, 5, 6, 7: Tables S3–S6).

The number of DA signatures detected by a particular method in each dataset ranged from a small minority to well over 50% of tested genera. For dataset 1, the maximum number of DA signatures detected by a particular method was 346 (baySeq, unfiltered data, encompassing 78% of tested genera), the minimum detected was 5 (ALDEx2 t-test, unfiltered data, encompassing 1% of tested genera), and the mean per method was 73 ± 95 for unfiltered data (encompassing on average 16% of tested genera) and 33 ± 18 for filtered data (encompassing on average 25% of tested genera) (Additional files 4, 6: Tables S3, S5). For dataset 2, the maximum number of DA signatures detected by a particular method was 424 (baySeq, unfiltered data, encompassing 76% of tested genera), the minimum detected was 11 (ANCOM, filtered data, encompassing 6% of tested genera), and the mean per method was 126 ± 133 for unfiltered data (encompassing on average 22% of tested genera) and 50 ± 23 for filtered data (encompassing on average 26% of tested genera) (Additional files 5, 7: Tables S4, S6). Methods on average detected a higher number of DA signatures in the larger, deeper sequenced dataset 2 (unfiltered data mean difference = 53, filtered data mean difference = 17) although the difference was only found statistically significant for filtered data [*t*_(41)_ = -2.9, *P* = 6E−3; Additional file 1] due to large variances seen with unfiltered data [*t*_(38)_ = -1.5, *P* = 0.13; Additional file 1]. No significant difference was found between datasets when the DA signature count for each method was normalized by the number of genera tested in the analyses [*t*_(30)_ = − 0.3, *P* = 0.74; Additional file 1].

Overall, despite differences in the size of datasets and heterogeneity in microbiome composition, no significant differences were observed between datasets in the proportion of genera being detected as differentially abundant on average. The number of detected DA signatures per method was significantly increased in dataset 2, but that is to be expected since it is a larger, more powered dataset both in sample size and number of genera detected.

### Concordance of DA signatures between methods and its relationship to proportion of genera detected as differentially abundant in unfiltered taxonomic data

**Fig. 1:**
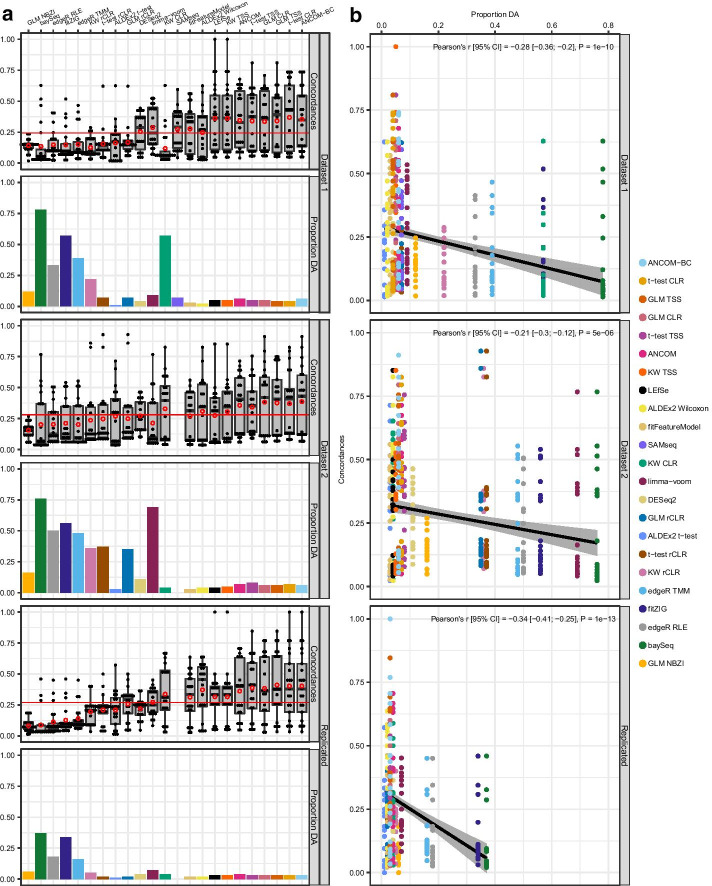
Pairwise concordances and proportion of genera detected as differentially abundant. Differential abundance testing was performed for 445 genera in dataset 1 and 561 genera in dataset 2 using various DA methods. Pairwise concordances (proportion of detected DA signatures in common between two methods out of the total DA signatures detected for those methods) were then calculated for each pair of methods. Column **a** for each method, the distributions of pairwise concordances and proportion of genera detected as differentially abundant for dataset 1 (top row), dataset 2 (middle row), and for DA signatures that replicated across datasets (bottom row). **b** The relationship between pairwise concordances and the proportion of genera detected as differentially abundant. Each dot in the boxplots represents a method, plotted according to the concordance it had with the method on the *x*-axis (22 dots for each method in dataset 1 and 21 dots for dataset 2 and replicated due to SAMseq failing to run for dataset 2). The bottom, middle, and top boundaries of each box in the boxplots represent the first, second (median), and third quartiles of the concordances. The lines extending from the top and bottom of the box extend to points within 1.5 times the interquartile range. Points extending above the whiskers are outliers. Red circles indicate the mean concordance for a method. Horizontal red lines indicate the mean concordance for either dataset 1, dataset 2, or replicated signatures. For dot plots, each concordance value was plotted against the proportion of genera deemed differentially abundant by a method, and a linear trend line (black solid line) was fitted to the data. The grey area surrounding the trend line is the 95% CI of the fitted line. Pearson’s correlation coefficient (*r*) and corresponding *P* value (P) were calculated for each dot plot to test strength of the relationship. Concordances: pairwise concordances for each method; Proportion DA: proportion of genera detected as differentially abundant (DA) by a method; GLM: generalized linear model; CLR: centered log-ratio; KW: Kruskal–Wallis; TSS: total sum scaling (relative abundances); rCLR: robust centered log-ratio transformation with matrix completion; RLE: relative log expression; TMM: trimmed mean of M-values; NBZI: negative binomial zero-inflated

On average, DA signature calls between methods varied in concordance, with pairwise concordances between methods ranging from 1 to 100% similarity with the mean pairwise concordance being 24% for dataset 1 and 28% for dataset 2 (Fig. 1a, top and middle rows; Additional file 8: Table S7A, B). For both datasets, results from baySeq, GLM NBZI, fitZIG, edgeR, ALDEx2 t-test, and all instances that utilized the rCLR transform consistently had the lowest pairwise concordances on average with other methods (12–27%). Results from Kruskal–Wallis with TSS, t-test and GLM with TSS and CLR, fitFeatureModel, ALDEx2 Wilcoxon, ANCOM, LEfSe, and ANCOM-BC consistently had the highest pairwise concordances on average with other methods (28–39%) especially among one another (40–64%). Methods that consistently resulted in lower concordances seemed to have, on average, detected a higher proportion of genera as differentially abundant compared to higher concordant methods (lower concordance mean = 0.3–0.4, higher concordance mean = 0.04–0.06; Fig. 1a, top and middle rows; Additional files 4, 5: Tables S3 and S4). Indeed, when correlating concordances with proportion of genera detected as differentially abundant, both dataset 1 and 2 showed a moderate, but highly significant, negative correlation where concordances decreased as a higher proportion of genera were called differentially abundant (Fig. 1b, top and middle rows), showing that this may be factor in the varying degrees of concordances observed here, and may reflect tendencies of some methods to detect larger amounts of false positives, at least in unfiltered data.

No significant difference between datasets was found for average pairwise concordances [*t*_(40)_ = − 1.5, *P* = 0.14; Additional file 1], however, some individual methods seemed to have dataset dependent influences on their concordances, notably Kruskal–Wallis with CLR and limma-voom (Fig. 1a, top and middle rows; Additional file 8: Table S7A, B). For dataset 1, Kruskal–Wallis with CLR resulted in the lowest mean concordance (12%) while it resulted in an above average mean concordance in dataset 2 (33%). This might be due to dramatic differences in the proportions of genera detected as differentially abundant between datasets (0.6 in dataset 1 versus 0.04 in dataset 2; Fig. 1a, top and middle rows; Additional files 4, 5: Tables S3 and S4). The opposite scenario was observed for limma-voom, where it had an above average mean concordance in dataset 1 (29%), but a below average mean concordance in dataset 2 (21%), which again might be due to differences in proportions of differentially abundant genera between datasets 1 and 2 (0.09 in dataset 1 vs 0.69 in dataset 2). These provide examples of how even the same method can behave differently depending on the underlying data being analyzed.

When calculating concordances for DA signatures that were replicated across datasets (Fig. 1a, bottom row; Additional file 8: Table S7C), the mean pairwise concordance between all methods stayed relatively the same compared to individual datasets with no statistically significant change in overall mean concordance (27%, *P* > 0.4). As seen in individual datasets, baySeq, GLM NBZI, fitZIG, edgeR, ALDEx2 t-test, and all instances that utilized the rCLR transform consistently had the least similar calls on average with other methods (8–26%), and Kruskal–Wallis with TSS, t-test and GLM with TSS and CLR, fitFeatureModel, ALDEx2 Wilcoxon, ANCOM, LEfSe, and ANCOM-BC consistently had the most similar calls on average with other methods (27–41%), and among each other (48–64%). In addition, Kruskal–Wallis with CLR and limma-voom, who were observed to have dataset specific effects on concordances and proportion of differentially abundant genera, resulted in above average mean concordances for replicated DA signatures. As expected, proportions of differentially abundant genera were reduced for replicated DA signatures, however, the same negative correlation observed in individual datasets between concordances and proportion of differentially abundant genera held true for replicated DA signatures (Fig. 1b, bottom row). This indicates that concordances (at least how they are being calculated here) overall seem to hold steady when calculating them for DA signatures detected in a single dataset or detected across datasets.

### Effect of taxonomic data filtering on DA method results and concordances

**Fig. 2:**
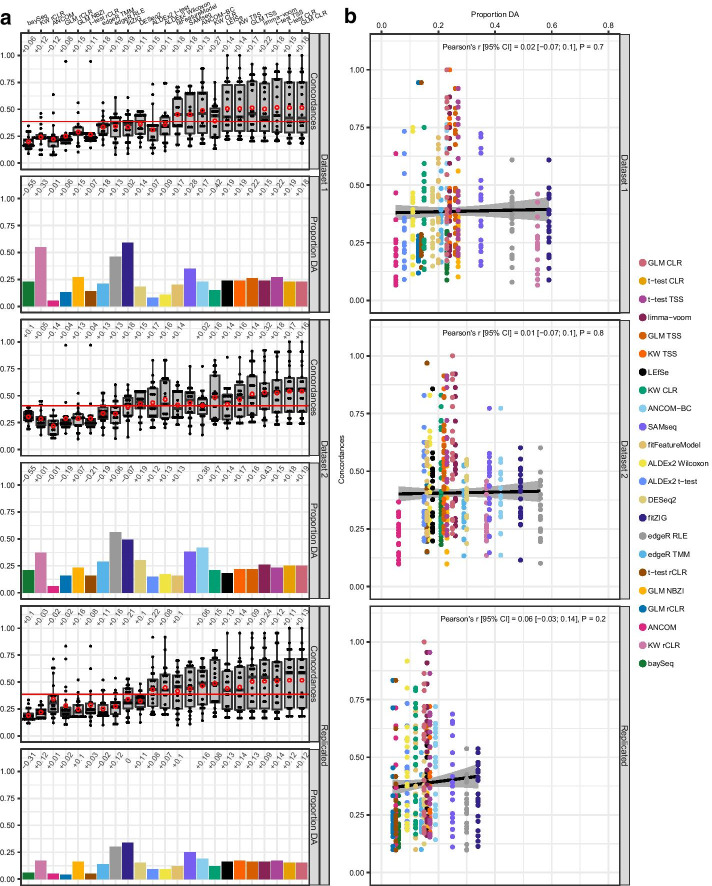
Effect of taxonomic data filtering on pairwise concordances and proportion of genera detected as differentially abundant. Differential abundance testing and calculation of pairwise concordances were performed again for 133 genera in dataset 1 and 195 genera in dataset 2 after filtering out genera that were found in < 10% of samples. Column **a** for each method, the distributions of pairwise concordances and proportion of genera detected as differentially abundant for dataset 1 (top row), dataset 2 (middle row), and for DA signatures that replicated across datasets (bottom row). Column **b** the relationship between pairwise concordances and the proportion of genera detected as differentially abundant. Each dot in the boxplots represents a method, plotted according to the concordance it had with the method on the *x*-axis (22 dots for each method). The bottom, middle, and top boundaries of each box in the boxplots represent the first, second (median), and third quartiles of the concordances. The whiskers (lines extending from the top and bottom of the box and ending in horizontal cap) extend to points within 1.5 times the interquartile range. The points extending above the whiskers are outliers. Red circles indicate the mean concordance for a method. The horizontal red line indicates the mean concordance for either dataset 1, dataset 2, or replicated signatures. Values above the box and whiskers are the differences in mean concordance between filtered and unfiltered (Fig. 1) data. Values above the bars in bar plots are the differences in proportion of differentially abundant genera between filtered and unfiltered (Fig. 1) data. For dot plots, each concordance value was plotted against the proportion of genera deemed differentially abundant by a method, and a linear trend line (black solid line) was fitted to the data. The grey area surrounding the trend line is the 95% CI of the fitted line. Pearson’s correlation coefficient (*r*) and corresponding *P* value (P) were calculated for each dot plot to test strength of the relationship. Concordances: pairwise concordances for each method; Proportion DA: proportion of genera detected as differentially abundant (DA) by a method; GLM: generalized linear model; CLR: centered log-ratio; KW: Kruskal–Wallis; TSS: total sum scaling (relative abundances); rCLR: robust centered log-ratio transformation with matrix completion; RLE: relative log expression; TMM: trimmed mean of M-values; NBZI: negative binomial zero-inflated

To examine the effects of data filtering on method results and concordances, pairwise concordances were calculated for DA testing results for filtered data (Additional files 6, 7: Tables S5 and S6). Overall, for both datasets, mean concordances per dataset improved by 13–15% and individual methods improved by 2–32% when DA testing was performed on filtered data (Fig. 2a, top and middle rows; Additional file 8: Table S7D, E). The only exception was ANCOM whose mean concordance decreased by 12% in dataset 1 and 14% in dataset 2, placing it among the consistently lower concordant methods, which were again baySeq, GLM NBZI, fitZIG, edgeR, and all instances that utilized the rCLR transform, but not ALDEx2 t-test as it’s mean concordance rose to above average for dataset 2 and replicated DA signatures. The group of higher concordant methods also remained the same, but with the addition of limma-voom and Kruskal–Wallis with CLR who had some of the largest improvements in mean concordance (+ 22% in dataset 1 and + 37% in dataset 2 for limma-voom; + 27% in dataset 1 and + 16% in dataset 2 for Kruskal–Wallis with CLR), and SAMseq, who failed to run for unfiltered dataset 2, but successfully ran for both filtered datasets.

Although members of lower and higher concordant method groups remained relatively the same, the relationship observed with unfiltered data between concordances and proportion of genera detected as differentially abundant was abolished by performing DA testing on filtered data (Fig. 2b, top and middle rows; correlation *P* > 0.7). This was most likely due to multiple factors including an overall increase in the proportion of differentially abundant genera being detected by higher concordant methods, a large decrease in the proportion of differentially abundant genera of lower concordant methods such as baySeq (− 0.6 in both datasets) and edgeR TMM (− 0.2 in both datasets), and a leveling out of limma-voom and Kruskal–Wallis with CLR proportions, potentially reflecting a reduction in dataset specific variation with removal of rarer genera.

These results show that, overall, concordances between methods increased when rarer genera were removed before testing, which might be due to a number of factors including minimization of noisy data, enriching datasets for more prevalent and stable genera that are more robust to method and dataset specific differences, providing a more stable denominator for data transformations and normalizations, and narrowing the pool of tested genera, which would automatically help reduce false positives. Observations noted for concordances and proportions of differentially abundant genera for datasets 1 and 2 held true for replicated DA signatures as well (Fig. 2a, b, bottom row; Additional file 8: Table S7F), showing that the supposed benefits of data filtering on concordances and differentially abundant genera proportions mentioned above translates to replication of DA signatures as well.

### Differentially abundant genera as a function of mean relative abundance and effect size

**Fig. 3:**
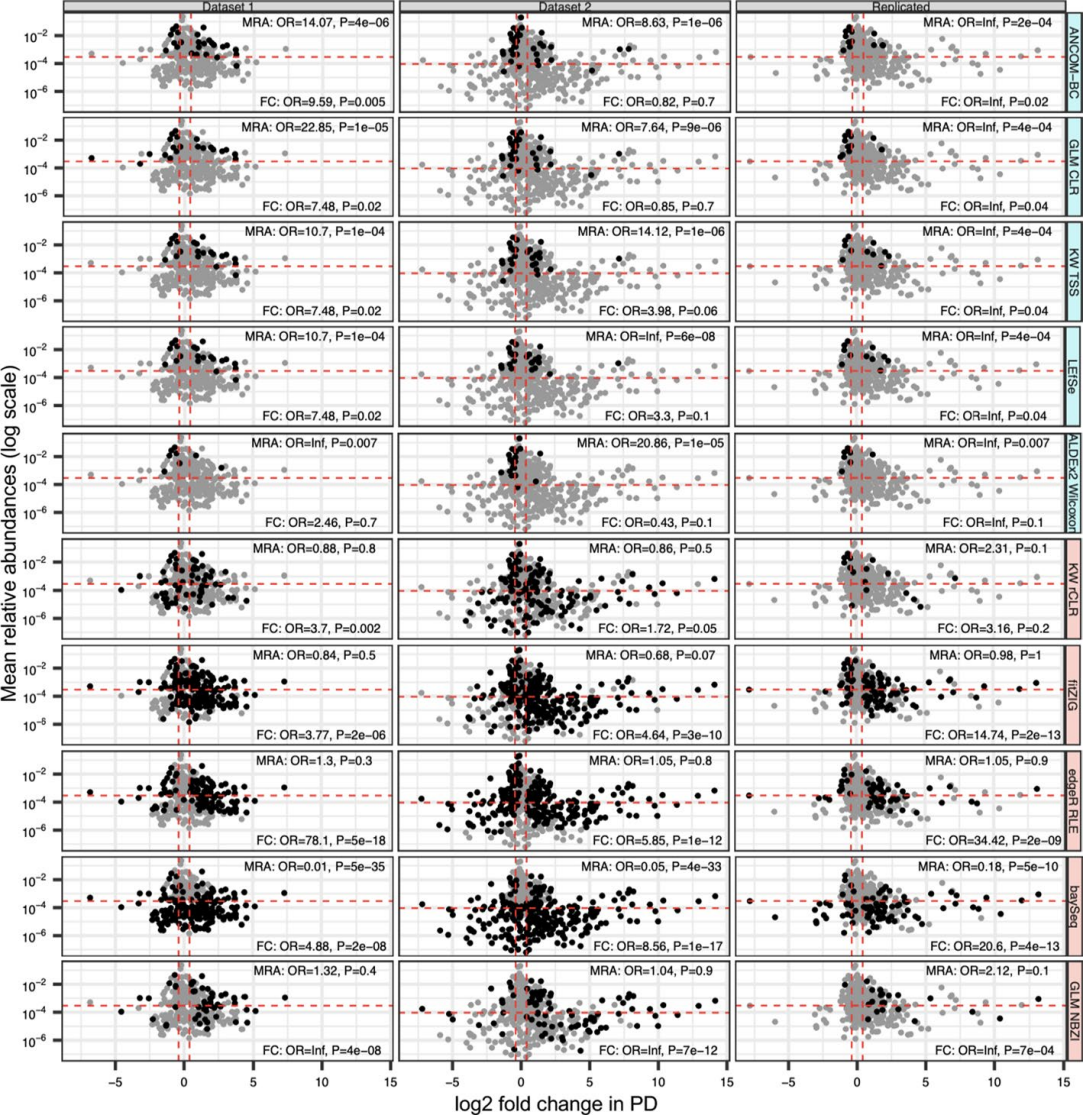
Detection of differentially abundant genera as a function of mean relative abundance and effect size. To observe what type of differentially abundant genera were being detected by methods based on mean relative abundance and effect size, the mean relative abundances of tested genera (on log scale) and log2 fold change of genera in PD were plotted for dataset 1 (left column), dataset 2 (middle column), and replicated signatures (right column), and repeating these plots for each DA method (rows). Fisher’s exact test was used to determine if DA signatures were enriched for more or less common genera, and genera with absolute fold change of 1.3 or higher. Individual points in the plots represent each tested genus, and is plotted according to that specific genus’ mean relative abundance in cases and controls and log2 fold change in PD. Composite mean relative abundances and fold changes were used to plot points for replicated DA signatures by taking the average between datasets. For each method, points are shaded black if a method detected a particular genus as differentially abundant, and grey if not detected as such. Horizontal dashed lines represent the median mean relative abundance for either dataset 1, dataset 2, or replicated signatures. Vertical dashed lines represent a fold change of ~ 1.3 in positive and negative directions. Blue labeled methods are methods that were found to have consistently higher than average concordances in both datasets and replicated signatures, while red labeled methods were found to have consistently lower than average concordances. For plot clarity, a representative five methods were chosen from each group to display for this figure. Full results for all methods can be found in Additional file 10: Fig. S1, and full results for all methods with filtered data can be found in Additional file 11: Fig. S2. MRA: mean relative abundance, results from Fisher’s exact test testing enrichment of more or less common genera in detected DA signatures; FC: fold change, results from Fisher’s exact test testing enrichment of genera with absolute fold changes > or < 1.3 in detected DA signatures; OR: odds ratio; P: *P* value of OR; GLM: generalized linear model; CLR: centered log-ratio; KW: Kruskal–Wallis; TSS: total sum scaling (relative abundances); rCLR: robust centered log-ratio transformation with matrix completion; RLE: relative log expression; NBZI: negative binomial zero-inflated

To observe what type of differentially abundant genera were being detected by DA methods based on mean relative abundance (MRA) and effect size, the MRAs of tested genera (on log scale) and log2 fold change of genera in PD were plotted against each other to view DA signatures of each method as a function of MRA and effect size (Fig. 3, Additional file 10: Fig. S1). Fisher’s exact test was then used to determine if DA signatures of each method were enriched for more, or less, common genera (defined as above or below the MRA median of each dataset and replicated DA signatures), and/or genera that had an effect size above a certain threshold (absolute fold change of ~ 1.3 or greater in PD).

For unfiltered data, obvious patterns for MRA were found between DA methods previously labeled as higher concordant methods versus those that were labeled lower concordant methods. When testing enrichment of DA signatures based on MRA, all DA signatures for higher concordant methods in both datasets were enriched for more common genera (above MRA median of each dataset) as all of these methods resulted in a significant Fisher’s exact test odds ratio (OR) well above 1 (Fig. 3, left and middle columns, first five rows; Additional file 10: Fig. S1, left and middle columns, first ten rows). More strikingly is that all ORs for replicated DA signatures of higher concordant methods resulted in an infinite value, meaning that all genera replicated by higher concordant methods had a MRA above the median (Fig. 3, right column, first five rows; Additional file 10: Fig. S1, right column, first ten rows). This suggest that higher concordant methods are overwhelmingly detecting more prevalent genera as differentially abundant, and exclusively replicating such. In contrast to this, lower concordant methods were found to have enrichment in less common genera (below MRA median; OR < 1), or no enrichment in either direction as genera above and below the MRA median were being detected and/or replicated as differentially abundant (OR ~ 1) (Fig. 3, bottom five rows; Additional file 10: Fig. S1 bottom nine rows). The only exception to this was ALDEx2 t-test who had same direction of ORs as the higher concordant methods (OR > 1). When looking at MRAs versus log2 fold change in PD of filtered data, DA signatures overall seemed to span MRAs above and below the median for the majority of methods, which makes sense as the data has already been enriched for more common genera (Additional file 11: Fig. S2).

Unlike MRA, there were less obvious patterns observed for detection of DA signatures at an absolute fold change threshold of 1.3 (Fig. 3, Additional file 10: Fig. S1). For higher concordant methods, there seemed to be a dataset specific influence on whether or not genera with absolute fold changes of 1.3 or greater were being preferentially detected (Fig. 3, left and middle columns, first five rows; Additional file 10: Fig. S1, left and middle columns, first ten rows). The ORs for dataset 1 were consistently higher, and usually more significant, than those of dataset 2, and were exclusively greater than 1, suggesting that the majority of detected genera’s fold changes were past the implemented threshold. A subset of ORs for dataset 2 were < 1, suggesting that these methods were detecting a higher fraction of DA signatures under the fold change threshold. Whether or not the detection of genera with fold changes below the implemented threshold in dataset 2 is due to increased power in the larger dataset, or increased false positives, remains to be seen. Again, like MRAs, the ORs for fold changes of genera being replicated in both datasets as differentially abundant were values of infinite, suggesting that only genera with an absolute fold change past the threshold were being replicated (Fig. 3, right column, first five rows; Additional file 10: Fig. S1, right column, first ten rows). This observation held true for some of the lower concordant methods, but not all, as some methods detected genera above, and below, the implemented fold change threshold in both datasets 1 and 2 (Fig. 3, left and middle columns, bottom 5 rows; Additional file 10: Fig. S1, left and middle columns, bottom 9 rows). However, although not as resolute as the infinite values seen with higher concordant methods, all ORs for replicated DA signatures of lower concordant methods were positive, again, suggesting that the majority of genera with absolute fold change past the threshold were being replicated (Fig. 3, right column, bottom five rows; Additional file 10: Fig. S1, right column, bottom nine rows). When looking at MRAs versus log2 fold change in PD of filtered data, the majority of genera detected by methods as differentially abundant had absolute fold changes greater than the implemented threshold (all ORs > 1; Additional file 11: Fig. S2), which suggests any ORs < 1 observed with unfiltered data might have been driven by detection of rarer genera.

These results suggest that methods belonging to the higher concordant group of methods are more likely to detect more common genera as differentially abundant, while methods belonging to the lower concordant group either err on the side of less common genera, or did not show a bias based on MRA. For fold changes, dataset specific effects seemed to be at play for higher concordant methods, and some lower concordant methods, but the majority of replicated DA signatures passed the absolute fold change threshold of 1.3.

### Hierarchical clustering of genera and methods based on similarities in DA signatures

**Fig. 4:**
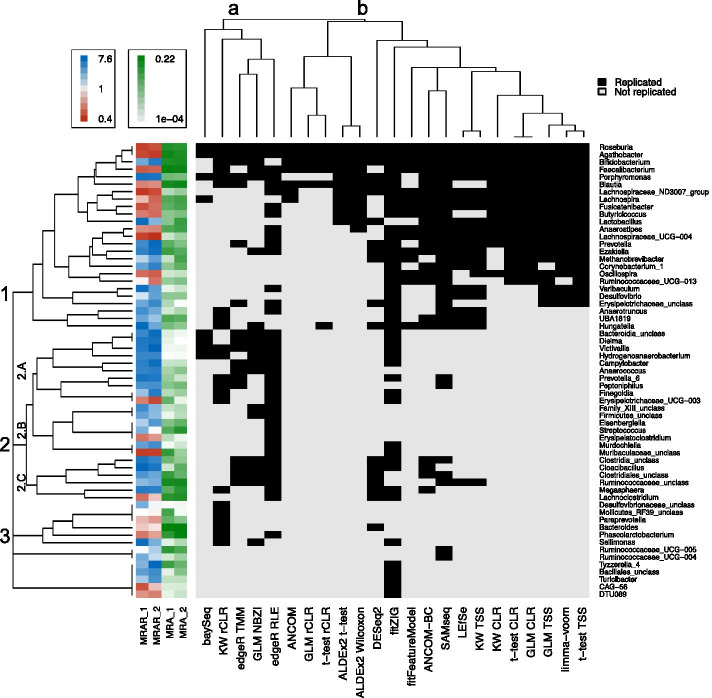
Hierarchical clustering of genera and methods based on similarity in replicated differential abundance signatures. Hierarchical clustering was performed to group genera (rows) and methods (columns) based on similarities in replicated differential abundance signatures and was visualized via heatmap. Three groups of genera were revealed by hierarchical clustering: (**1**) genera more likely to be called differentially abundant by the majority of methods in both datasets, (**2**) genera who were mostly found enriched in PD and called differentially abundant in both datasets by a subset of methods, and (**3**) genera who were called differentially abundant by only 1–3 methods. Group 2 interestingly contained a sub-group of rarer genera with larger effect sizes (2.A) compared to other group 2 sub-groups (2.B and 2.C) and groups 1 and 3. All but one of these genera were enriched in PD and detected almost exclusively by methods who consistently resulted in lower than average concordances. Two groups of methods were also revealed: methods that mainly replicated DA signatures of group 2 (**a**), and the remaining methods (**b**). Hierarchical clustering was based on method results from filtered taxonomic data, and only genera that were detected as differentially abundant in both datasets by at least one method were included in the clustering and heatmap (61 in total). Cells correspond to a differential abundance signature that was replicated across datasets (value = 1, color = black), or was not replicated (value = 0, color = grey). Mean relative abundance ratios for genera in dataset 1 (MRAR_1) and dataset 2 (MRAR_2) were plotted next to the heatmap, and given a color gradient from red (lowest MRAR) to light grey (MRAR ~ 1) to blue (highest MRAR). Mean relative abundances of genera for dataset 1 (MRA_1) and dataset 2 (MRA_2) were also plotted next to the heatmap, and given a color gradient from light grey (lowest MRA) to dark green (highest MRA). GLM: generalized linear model; CLR: centered log-ratio; KW: Kruskal–Wallis; TSS: total sum scaling (relative abundances); rCLR: robust centered log-ratio transformation with matrix completion; RLE: relative log expression; NBZI: negative binomial zero-inflated

To observe what groups of DA signatures either all, or subsets, of methods were converging upon, hierarchical clustering of DA signatures and methods was performed to group each based on similarities in DA signature replications (Fig. 4). To increase clarity of the hierarchical clustering and visualization using heatmap, results from filtered data were used and only genera that were replicated by at least one method as being differentially abundant in both datasets were included in the clustering and heatmap. Hierarchical clustering grouped DA signatures into three groups. The first group encompassed 25 genera (19% of genera tested and in common between datasets) that were more likely to be detected as differentially abundant across methods in both datasets (13 ± 5 methods on average; Fig. 4, group 1; Additional file 9: Table S8). This group included genera that were found to be both enriched or depleted in PD, and had a wide range of MRAs and effect sizes ranging from highly prevalent genera with moderate effect sizes (e.g. *Agathobacter,* MRA = 0.01–0.03, absolute fold change = 1.8–1.9) to less common genera with larger effect sizes (e.g. *Ezakiella*, MRA = 3E−3–5E−3, absolute fold change = 3.3). The second group included 23 genera (18% of genera tested and in common between datasets) that were largely enriched in PD and detected as differentially abundant in both datasets by a subset of methods (4 ± 2 methods on average; Fig. 4, group 2; Additional file 9: Table S8). Group 2 also included genera with a range of fold changes and MRAs (mean absolute fold change = 2.7–3.1, mean MRA = 2E-3 – 3E-3), but contained a sub-group of genera that were of interest (Fig. 4, sub-group 2.A). Genera in this sub-group contained only genera that were enriched in PD (barring one), had higher effect sizes than other groups (mean absolute fold change = 3.1–3.7), was made up of genera with very low MRAs (mean MRA = 6E−4–9E−4), and were largely detected in both datasets by a subset of methods that were included in the lower concordant group of methods previously mentioned above (Fig. 4, sub-group 2.A; Additional file 9: Table S8). The third group included 13 genera (10% of genera tested and in common between datasets) who were only replicated by one to a few method(s) (Fig. 4, group 3; Additional file 9: Table S8).

Hierarchical clustering of methods revealed two groupings of methods. The first group contained more than half of the lesser concordant methods (baySeq, Kruskal–Wallis with rCLR, edgeR TMM, edgeR RLE, GLM NBZI; Fig. 4, group A; Additional file 9: Table S8) that, together, detected the majority of genera included in sub-group 2.A as differentially abundant across datasets. The second group contained the remainder of methods (Fig. 4, group B; Additional file 9: Table S8). The next major split in the method dendrogram was between the remaining methods and ANCOM, t-test and GLM with rCLR, and ALDEx2 who had the lowest number of replicated DA signatures (8 ± 3). No other groupings of methods seemed obvious from the hierarchical clustering, but of note, the next major split in the dendrogram for group B was DESeq2, then fitZIG, then the higher concordant methods (ANCOM-BC, SAMseq, LEfSe, Kruskal–Wallis with TSS and CLR, fitFeatureModel, t-test with TSS and CLR, limma-voom, and GLM with TSS and CLR), which made up the majority of the methods that replicated DA signatures in group 1.

## Discussion

In summary, various differential abundance testing methods were found in the literature and used to detect differentially abundant genera in PD patients versus healthy controls in two large PD-gut microbiome datasets. Methods spanned multiple fields and had both common and unique characteristics when compared to one another. Differential abundance signatures were detected by all methods and the number of DA signatures detected by each method ranged from a small subset of genera to over half of the genera tested. Overall, 78–92% of genera tested were found to be differentially abundant by at least one method in each dataset, while 5–22% were called differentially abundant by the majority of methods (depending on dataset and filtering of taxonomic data prior to testing). Pairwise concordances between method calls varied overall, but were improved by using taxonomic data that had rarer genera removed before DA testing was performed. Regardless of whether unfiltered or filtered taxonomic data was being analyzed, certain methods consistently resulted in higher mean pairwise concordances (e.g. ANCOM-BC, LEfSe), while others consistently resulted in lower mean pairwise concordances (e.g. edgeR, fitZIG). Higher concordant methods tended to detect more common genera as differentially abundant while lower concordant methods either did not have an obvious preference, or erred toward less common genera. The relationship between detected DA signatures and fold change of genera in PD was less obvious, but there seemed to be a dataset effect for higher concordant methods, and some lower concordant methods, where more genera with absolute fold change < 1.3 were detected in dataset 2. For all methods, the majority of genera being replicated as differentially abundant in both datasets had absolute fold change of 1.3 or greater. Hierarchical clustering revealed three groups of DA signatures that were (1) replicated by the majority of methods on average and included genera previously associated with PD, (2) replicated by a subset of methods and included genera largely enriched in PD, and (3) replicated by one to a few method(s). Although datasets were heterogeneous in microbiome composition, no significant differences were observed between datasets in average concordances and the proportion of genera being detected as differentially abundant on average.

The variation between method results reported here aligns with the variation between differential abundance testing method performances previously reported in method comparison studies [3–5]. Performance of methods could not be assessed here, as analyses were conducted on real datasets where the true answers are unknown, but it was observed that certain methods consistently resulted in above average pairwise concordances, and even higher pairwise concordances among each other, while other methods consistently resulted in lower than average pairwise concordances. Interestingly, methods found here to have above average pairwise concordance were also previously reported to have lower FPR and/or FDR (less than 0.1; e.g. ANCOM-BC, Kruskal–Wallis, t-test, ALDEx2, fitFeatureModel, ANCOM; Additional file 2: Table S1). This, along with observations made in the current study, might help explain why they resulted in such high concordances with each other. Methods with lower FPR/FDR have been previously reported to be conservative in their performance [3], detecting less taxa as differentially abundant compared to higher FPR/FDR methods (which was also observed here). In this study, these methods seemed to detect DA signatures across datasets that included more prevalent genera (i.e. the relationship between detected genera and MRA shown in Fig. 3) and were robust to inter-methodological variation (i.e. genera shown in Fig. 4, group 1 that were more likely to be replicated by the majority of methods on average). Taken together, we can extrapolate that methods with lower FPR and/or FDR might be more likely to converge on the same taxa because they are detecting less taxa as differentially abundant overall, and the signatures they do detect are more prevalent and stable members of the microbial community that are potentially more robust to methodological and population variation. Based on results of this study, these methods seem to be good candidates for detection of DA signatures that will be more resistant to inter-study variation in DA method choice. This comes with the caveat that some true associations might be missed as these methods seem to be detecting more “high confidence” hits, and therefore, might be conservative as previously stated. In contrast, some methods found here to have below average pairwise concordance were also previously reported to have higher FPR and/or FDR (greater than 0.13; e.g. baySeq, edgeR, fitZIG, GLM NBZI; Additional file 2: Table S1). In this study, lower concordant methods overall detected a higher proportion of genera as differentially abundant compared to higher concordant methods, which undoubtedly plays a role in their higher FPR/FDR. However, this is not the only factor as was shown in this study when the relationship between concordances and proportion of differentially abundant genera was removed with data filtering, yet members of the lower concordant group of methods still remained constant for the most part.

A surprising finding from this study was the effect of input data filtering prior to DA testing, but intuitively, it makes logical sense that removal of rarer taxa increases the concordance between method results. Minimizing the pool of taxa available for detection will automatically help remove some potential false positives, especially for methods such as baySeq and fitZIG that detected a large amount of rarer genera as differentially abundant. Filtering data also lowers the multiple testing correction burden, so more conservative methods, such as the high concordant methods of this study, will detect a higher proportion of taxa as differentially abundant. This, in conjunction with reduced proportion of differentially abundant taxa for more liberal methods, will bring method results more into alignment with one another as was observed in this study. However this was not the case for all methods as shown with ANCOM. Filtering of input data before analysis actually decreased the number of detected DA signatures for ANCOM. This might be due to the different statistics used by ANCOM compared to the standard FDR q-value used by the other methods included in this study. To determine significance, ANCOM calculates a *W* statistic, which is the number of times the log-ratio of a taxon with every other taxon being tested was detected to be significantly different across groups (in this case PD versus control) [6]. Because *W* statistics are based on pairwise comparisons between all taxa being tested, they will automatically decrease overall if less taxa are included in the analysis, and the threshold range for significant *W* statistics will also decrease. In addition, if low prevalent taxa are being removed, this will not only decrease the *W* statistics overall, but now *W* statistic calculation might become more conservative since more prevalent, and potentially more stable, taxa have been selected for, the ratios of which might not differ enough to be detected as significant at a particular *W* statistic threshold. This could potentially make the analysis overly conservative (as seen in this study), therefore, if using ANCOM, it might be beneficial to perform ANCOM using the full surveyed microbiome, removing none, or only the very rare taxa, before analysis. This effect was not observed for the more recently updated ANCOM-BC, as ANCOM-BC uses a linear regression framework to perform DA testing without the use of pairwise log-ratio comparisons and calculation of *W* statistics.

A finding from this study that helped illustrate the behavior of the methods on the data analyzed here was the detection of two groups of genera that were converged upon by either the majority or subset of methods. Hierarchical clustering of genera based on similarity in DA signature replication showed one group of genera that were more likely to be detected as differentially abundant in both datasets by the majority of methods on average (Fig. 4, group 1). Theoretically, this group might be looked at as the “high confidence” group, as methods from across the spectrum tended to detect the genera in this group as differentially abundant in both datasets, especially those methods in the higher concordant group. This group included genera previously associated with PD such as *Bifidobacterium*, *Lactobacillus*, and short-chain fatty-acid producing bacteria *Faecalibacterium*, *Roseburia*, *Blautia*, and other members of the *Ruminococcaceae* and *Lachnospiraceae* family [22, 23]. It also included members of a correlated poly-microbial group of genera found previously to be increased in PD (*Porphyromonas*, *Corynebacterium_1*, *Prevotella*, *Ezakiella*, *Varibaculum*) [21]. Hierarchical clustering also revealed a second group of genera that were only detected as differentially abundant in both datasets by a subset of methods, and contained an interesting sub-group that contained mostly genera enriched in PD that had low control MRAs, and higher effect sizes on average compared to the rest of group 2, and groups 1 and 3 (Fig. 4, sub-group 2.A; Additional file 9: Table S8). Without the use of these methods, this group of genera might have been missed, arguing that, although some of these methods were previously reported to have higher FPR/FDR [3–5], they might detect DA signatures that may be missed by more conservative methods. It seems these methods should be used with caution, however, as stated earlier they tended to detect a large portion of genera as differentially abundant, and may replicate false positives by chance just from the sheer volume of differentially abundant taxa being detected.

One of the biggest limitations of this study is the lack of ability to test the actual performance of these methods, as no simulations were performed and only real data was used, so the true answers are unknown. Luckily, the majority of the methods studied here have been previously subjected to comparison studies on simulated data, and results from those studies could be used to inform the discussion of the results from this study [2–5]. Unfortunately, not all methods implemented in this study had previously reported performance metrics (i.e. LEfSe and Kruskal–Wallis, t-test, and GLM with CLR and rCLR), therefore, it is difficult to speculate reasons for why they resulted in higher or lower concordances with other methods. A methodological limitation of this study is the choice of parameters used for each method. Each method may contain multiple functions with multiple parameters, and it was beyond the scope of this study to try different combinations of parameters to fully optimize each method for the data being analyzed. Parameter choices were made based on what was default for the method and/or what was recommended for the method by the method authors especially in the context of microbiome data analysis, but this process is inherently biased as it was not attempted to try every combination of parameter choice possible for each method. Another methodological limitation of this study is the variation in normalization strategies used for different methods to account for inter-sample differences in total sequencing depth. Different normalization strategies have been previously shown to impact method performance [4], therefore, changes in normalization strategy for some methods might minimize or exacerbate differences seen here between method results. It was beyond the scope of this study to assess the effect of all available normalization strategies on DA method concordances, therefore, normalization strategies were chosen for methods based on if a built in normalization method was available within the method function itself, or the method’s R package, and recommended by method authors (i.e. calculation of scaling factors for DESeq2, edgeR, baySeq; CSS for metagenomeSeq methods; log-ratios for ALDEx2 and ANCOM; bias correction in ANCOM-BC; log2-CPM for limma-voom; SAM normalization for SAMseq; offset variable for GLM NBZI). If a DA method did not have a built-in transformation/normalization strategy (i.e. Kruskal–Wallis, Welch’s t-test, GLM), they were performed using two commonly implemented transformation/normalization strategies seen in the literature (i.e. TSS and the compositionally aware CLR), and additionally, a more recently described, compositionally aware transformation (robust CLR with matrix completion) [20]. Because this study was performed on two datasets collected for the study of a specific disease in a specific aged population with data derived from a specific host source (stool), the results reported here might not translate to all types of microbiome datasets. Both datasets analyzed here were also created using uniform methodology, therefore, results may also change if attempting to analyze two datasets created using different methodologies. The genera detected in this study as differentially abundant between PD and control subjects should be interpreted with caution as the goal of this study was to compare the results of different DA methods when performed on microbiome datasets of real, complex disease, and did not take into account any confounding variables that might drive false signals. Because no confounders were taken into account during the analyses of this study (e.g. PD medications, constipation, age, sex, diet), differentially abundant genera detected in this study cannot be reported as truly “associated” with PD as the proper steps to guard against false positives due to underlying study population characteristics were not taken. Still, majority of genera listed as part of group 1 have been previously reported as associated with PD [21], and genera listed as part of sub-group 2.A provide interesting findings that will hopefully be elucidated further in future investigations.

Although true performance of methods could not be assessed in this study due to the lack of ground truth for detected DA signatures, comparisons performed here revealed some potential strengths and shortcomings of individual methods, at least when performed on data similar to what was used in this study. An obvious strength for some of the methods used here was the ability to detect and replicate DA signatures that were more concordant with other methods on average regardless of dataset and whether unfiltered or filtered data was used as input. These methods include ANCOM-BC, GLM with CLR and TSS, t-test with CLR and TSS, Kruskal–Wallis with TSS, and LEfSe as they were consistently included in the higher concordant group of methods regardless of the scenario. For detection of DA signatures that will be the most resistant to methodological and inter-study variation, ideally one would choose multiple DA methods to implement and assess their concordances, but if needing to choose one DA method, the recommendation based off the present, and previous, comparison studies would be to choose method(s) from the ones stated above that take compositionality of data into account (ANCOM-BC and/or GLM or t-test with CLR). Based on comparisons performed in this study, t-test with CLR seems to have the overall highest and most stable concordances of the three, but if needing to include covariates and more complex linear models, ANCOM-BC and GLM with CLR are valid choices, and will provide statistics and *P* values for further post analyses such as meta-analysis. Kruskal–Wallis with CLR would be assumed a good choice since t-test is a parametric test and CLR transformation does not always address non-normality of the data fully, but a blatant shortcoming of using Kruskal–Wallis with CLR was observed in this study. Concordances on average were found to be unstable for Kruskal–Wallis with CLR, being more influenced by dataset and data filtering compared to the other methods that utilized this transformation (t-test and GLM). This might be due to a difference in the geometric mean, used as the denominator in CLR, between PD and control subjects for some genera, which Kruskal–Wallis may be more sensitive to, and therefore, would have had a higher proportion of genera detected as differentially abundant (as seen for dataset 1, unfiltered data). Further investigation into this is warranted to elucidate the reasoning behind this instability. Another shortcoming was noted for the rCLR transformation with matrix completion when used with GLM, t-test, and Kruskal–Wallis. Concordances for methods using the rCLR transformation were consistently in the lower concordant group of methods, and showed dataset specific effects on the proportion of genera that were being detected as differentially abundant for unfiltered data. Additionally, very few DA signatures were replicated when using the rCLR transformation in both unfiltered and filtered data, which might be a sign of spurious DA signatures being detected. This might be due to the matrix completion step after the CLR transformation of non-zero components. While the matrix completion algorithm makes the best guess it can on what data should be filled in for missing data, it is still only a guess, and may introduce artifacts into the data. Also, keeping the non-missing data relatively the same, but providing imputed values for zero entries does not accurately recapitulate what occurs during sequencing if zeros present in the data are due to low sampling of the microbial community. If that is the case, then the existing values also need to be adjusted for the imputed input.

## Conclusion

In conclusion, various differential abundance testing methods were performed for two large PD-gut microbiome datasets and their results were compared. Results varied between methods, but some methods resulted in higher pairwise concordances, while others resulted in lower concordances. Filtering of genera before analysis improved concordances overall except for ANCOM, where filtering reduced the number of DA signatures detected, most likely due to the way *W* statistics are calculated and used for significance. The majority of methods converged on a group of DA signatures that seemed more robust to inter-methodological differences, while a subset of methods converged on a smaller sub-group of DA signatures involving genera mostly enriched in PD with low relative abundances. This study helps to fill a void in the literature on how different DA methods behave when performed on real, complex disease oriented gut microbiome datasets, and hopefully it will help inform future studies looking to perform these types of analyses, especially those working with gut microbiome data derived from stool for studying Parkinson disease.

## Methods

### Subjects, metadata, gut microbiome

The original study was approved by institutional review boards at all participating institutions. Subjects, metadata, and gut microbiome data of datasets 1 and 2 have been previously described [21, 24]. We enrolled subjects and collected metadata and stool samples from 212 PD and 136 neurologically healthy control subjects for dataset 1, and 323 PD and 184 neurologically healthy controls for dataset 2. Dataset 1 subjects were enrolled in Seattle, WA, Albany, NY, and Atlanta, GA, while all dataset 2 subjects were enrolled in Birmingham, AL. Methods for enrollment and collection of metadata and fecal samples were uniform across enrollment sites. PD was diagnosed according to the UK Brain Bank criteria by movement disorder specialists. Controls were self-reported free of neurological disease. Metadata were collected using questionnaires, or extracted from medical records. Stool samples were collected at home using DNA/RNA-free sterile swabs and mailed through U.S. postal service. All subjects provided written informed consent for their participation in the study.

DNA was extracted from stool samples using the automated MoBio PowerMag Soil DNA Isolation Kit (dataset 1) or manual MoBio PowerSoil DNA Isolation Kit (dataset 2). Hypervariable region 4 (V4) of the 16S rRNA gene was amplified with primers 515F-806R. Paired-end 150 bp (dataset 1) or paired-end 250 bp (dataset 2) sequencing was performed on V4 amplicons using Illumina MiSeq. Fifteen samples in dataset 1 resulted in low sequence count and were excluded.

Bioinformatic processing of sequences was performed separately for each dataset. Primers were trimmed from sequences using cutadapt v 1.16 [25]. DADA2 v 1.8 was used for quality trimming and filtering sequences, de-replicating sequences, inferring amplicon sequence variants (ASVs), merging of forward and reverse sequences, and detection and removal of chimeras [26]. Final ASV tables for dataset 1 and dataset 2 contained 6,844 unique ASVs for 201 PD and 132 controls samples and 12,198 unique ASVs for 323 PD and 184 control samples respectively. Taxonomy was assigned to ASVs using DADA2’s native implementation of the Ribosomal Database Project naïve Bayesian classifier with SILVA v 132 as reference and a bootstrap confidence of 80% [27]. Phylogenetic trees were constructed by first performing a multiple sequence alignment with DECIPHER v 2.8.1 [28], then building a phylogenetic tree with phangorn v 2.8.1 [29]. Phyloseq v 1.24.2 was used to create a phyloseq object for each dataset containing their respective ASV table, taxonomy classifications, phylogenetic trees, and subject metadata [30]. To agglomerate ASV level phyloseq objects to genus level, the tax_glom function in phyloseq was used without removal of unclassified genera. Total number of genera detected in dataset 1 was 445. Total number of genera detected in dataset 2 was 561. Total number of genera in common between both datasets was 380.

### Differential abundance testing

Differential abundance testing was performed in datasets 1 and 2 separately. Method characteristics and parameters chosen for each method that differed from default can be found in Additional file 2, 3: Tables S1 and S2 respectively. All methods were performed twice, once for all genera detected in datasets 1 and 2 (referred to as "unfiltered data"), and again, for genera present in at least 10% of samples (referred to as “filtered data”; 133 genera in dataset 1, 195 genera in dataset 2, and 129 genera in common between datasets) to observe effect of taxonomic data filtering on method results and comparisons. For methods that did not have a built-in transformation/normalization strategy (i.e. Welch’s t-test, Kruskal–Wallis rank-sum test, generalized linear model), three strategies were performed on genera abundances before testing: the commonly used TSS (also referred to as relative abundances), the more compositionally aware CLR transform [19], and a more recently described robust CLR transform with matrix completion using the OptSpace algorithm [20]. The TSS and CLR of each abundance count was calculated using the following formulas in R v 4.0.5:

$$\begin{aligned} & {\text{TSS}}: \, \left[ {X_{{{\text{taxa}}}} /{\text{ sum}}\left( {X_{1} ,X_{2} , \, \ldots X_{n} } \right) \, } \right] \\ & {\text{CLR}}: \, \left[ { \, \log \left( {X_{{{\text{taxa}}}} + \, 1} \right) \, {-}{\text{ mean}}\left( {{\text{log}}\left( {X_{1} + \, 1, \, X_{2} + \, 1, \ldots \, X_{n} + \, 1} \right)} \right) \, } \right] \\ \end{aligned}$$

where *X*_taxa_ is the raw abundance of the current genus whose abundances are being transformed, and *X*_1_, *X*_2_, … *X*_*n*_ are the raw abundances of every genus in the same sample as the current genus. The rCLR was calculated in the same manner as the CLR, but no pseudo-count of 1 was added, only non-zero components of the data were included, and the transform was followed by matrix completion of missing values using the OptSpace function in the R package ROptSpace v 0.2.2 [31]. When performing TSS for parametric methods (t-test and GLM), abundances were given a pseudo-count of 1, then log transformed before applying TSS, as this normalization method has no built in strategy for improving normality of the data. Remaining details for performing DA testing for all methods are as follows.

#### Analysis of compositions of microbiomes (ANCOM) [6]

Raw counts of genera were used as input to the ANCOM.main function from the ANCOM v 2 R code (downloaded from https://sites.google.com/site/siddharthamandal1985/research). PD vs control was specified as the main variable. The taxa-wise FDR option (multcorr = 2) was chosen for the multiple testing correction method. An FDR significance threshold of 0.05 was chosen for calculation of *W* statistics. *W* statistics greater than or equal to 80% of the total number of genera tested were considered significant.

#### Analysis of compositions of microbiomes with bias correction (ANCOM-BC) [7]

Raw genera counts were used as input to the ancombc function in the ANCOMBC R package v 1.0.5 specifying the p_adj_method to be “BH” and zero_cut threshold to be “1” (so ANCOM-BC itself would not perform any data filtering). All other options were left as default.

#### ALDEx2 [8]

Raw genera counts were used as input for the aldex function in ALDEx2 R package v 1.22.0 specifying 1000 Monte Carlo samples. Both Wilcoxon (ALDEx2 Wilcoxon) and t-test (ALDEx2 t-test) were used for testing differences in genera relative abundances between PD and controls. *P* values were corrected for multiple testing using BH FDR method implemented in the aldex function.

#### baySeq [9]

PD and control designations were used as the replicate structure. A list of two group structures was created where one group structure specified all subjects belonged to the same group, and the other specified PD and control groups. The replicate structure, list of group structures, and raw genera counts were combined into a countData object. Total sequence counts per sample were calculated and manually supplied to the countData object. Priors were estimated from a negative binomial distribution using the function getPriors.NB in baySeq R package v 2.14.0, then likelihoods were estimated using function getLikelihoods in baySeq. FDR values were calculated using the topCounts function in baySeq.

#### DESeq2 nbinomWaldTest [10]

Using raw genera counts, normalization factors were calculated using the function estimateSizeFactors in DESeq2 R package v 1.30.1 specifying type = “poscounts”. Testing for differential relative abundance between PD and controls was performed using the DESeq function in DESeq2 with default parameters. *P* values were corrected for multiple testing using BH FDR method implemented in the results function in DESeq2.

#### edgeR exactTest-TMM (edgeR TMM) [11]

Using raw genera counts, normalization factors were calculated with the TMM method using the calcNormFactors function in edgeR R package v 3.32.1. Common and tagwise dispersions were then estimated using estimateCommonDisp and estimateTagwiseDisp functions in edgeR. Testing for differential relative abundance between PD and controls was performed using exactTest function in edgeR. *P* values were corrected for multiple testing using BH FDR method implemented in the topTags function in edgeR.

#### edgeR exactTest-RLE (edgeR RLE) [11]

Using genera counts with a pseudo-count of 1 added, normalization factors were calculated with the RLE method using the calcNormFactors function in edgeR. The remaining steps were the same as edgeR TMM.

#### Generalized linear model (GLM)

A standard linear regression model using Gaussian distribution was fitted for the transformed abundances of each genus with the glm function from the R stats package specifying PD vs control as the independent variable. *P* values were calculated using the base summary function in R and corrected for multiple testing using BH FDR method implemented in the p.adjust function from stats package.

#### Kruskal–Wallis rank-sum test [12]

The kruskal.test function from the stats R package was used to test for significant differences in transformed genera abundances between PD and controls. *P* values were corrected for multiple testing using Benjamini-Hochberg (BH) FDR method implemented in the p.adjust function from stats package.

#### Linear discriminant analysis Effect Size (LEfSe) [13]

Genera counts were transformed using TSS. Sample IDs, case/control class designations, and genera relative abundances were exported from R and used as input for LEfSe v 1.0.8.post1 (downloaded using LEfSe bioconda recipe https://bioconda.github.io/recipes/lefse/README.html). Only genus level taxonomy designations were included in the LEfSe input. The LEfSe input was formatted using the lefse-format_input.py script specifying the normalization value to be “1E6”. LEfSe analysis was then ran on the formatted data using the run_lefse.py script with default parameters. Since LEfSe only outputs uncorrected *P* values for features that it finds significant, LEfSe analysis was ran again, but this time specifying parameters that would output all *P* values. The full range of LEfSe *P* values were multiple testing corrected using BH FDR method implemented in the p.adjust function from stats package. These corrected *P* values were substituted for the uncorrected *P* values outputted by the default LEfSe run.

#### limma-voom [14]

Using raw genera counts, TMM values were calculated using the calcNormFactors function in edgeR. Log2-CPM transformation and mean–variance trend estimation was performed using the voom function in limma R package v 3.46.0. Testing was performed by first fitting a linear model for each genus using function lmFit in limma, then testing for differential relative abundance between PD and controls using the eBayes function in limma. *P* values were corrected for multiple testing using BH FDR method implemented in the topTable function in limma.

#### metagenomeSeq zero-inflated Gaussian (fitZIG) [15, 16]

CSS was applied to genera counts using the cumNorm function in metagenomeSeq R package v 1.32.0. A zero-inflated Gaussian model was fitted for each genus using function fitZig in metagenomeSeq. *P* values were corrected for multiple testing using BH FDR method implemented in the MRfulltable function in metagenomeSeq.

#### metagenomeSeq fitFeatureModel [15, 16]

CSS was applied to genera counts using cumNorm function in metagenomeSeq. A zero-inflated log-normal model was fitted for each genus using function fitFeatureModel in metagenomeSeq. *P* values were corrected for multiple testing using BH FDR method implemented in the MRfulltable function in metagenomeSeq.

#### Negative binomial generalized linear model with and without zero-inflation (GLM NBZI)

Total sequence count was calculated for each sample. Using raw counts, a negative-binomial generalized linear model with and without a zero-inflation component was fitted for each genus with the glmmTMB R package v 1.0.2.1 using log(total sequence count) as an offset variable, and PD vs control as the independent variable. Results were extracted from the model with the lowest Akaike information criterion. *P* values were calculated using the base summary function in R and corrected for multiple testing using BH FDR method implemented in the p.adjust function from stats package.

#### SAMseq [17]

The SAM method for normalization of sequence counts (Anscombe transformation, then dividing by the square root of sequencing depth) was applied to genus counts using the samr.norm.data function in the samr R package v 3.0. Normalized values were rounded to the nearest integer. Normalized genera counts were used as input for the SAMseq function in the samr R package specifying “Two class unpaired” as the response type and the fdr.output as “1” in order to get full result list. FDR q-values were extracted from “siggenes.table” in the SAMseq output.

#### Welch’s t-test (t-test) [18]

The t-test function from the stats R package was used to test for significant differences in transformed genera abundances between PD and controls. *P* values were corrected for multiple testing using BH FDR method from the p.adjust function.

Unless otherwise mentioned above, significance was set at FDR < 0.05. Significant differences in a genus’ relative abundance between PD and control groups is referred to as a “DA signature” for the purpose of this manuscript. For a particular method, a DA signature was considered replicated in both datasets if it reached multiple testing corrected significance in both datasets for that method, and was found to have a PD to control mean relative abundance ratio (MRAR; also referred to as fold change) in the same direction for both datasets.

### Concordance of DA signatures across DA methods

To measure similarity in detected DA signatures between methods, pairwise concordances were calculated between each pair of methods. To calculate pairwise concordances between methods for each dataset individually, a binary genus by method matrix was first created with values denoting which methods did (1), or did not (0), detect a DA signature between a genus and PD. Then, for each pair of methods in turn, concordances were calculated by summing the number of detected DA signatures that were the same between both methods (both having 1 for a particular genus) and dividing by the total number of detected DA signatures between both methods. Calculating concordance in this manner gives the proportion of DA signatures in common between two methods out of the total detected DA signatures, providing a measure of how well two methods are hitting the same targets. To calculate pairwise concordances between methods for DA signatures that replicated across datasets, a binary genus by method matrix was first created with values denoting which methods replicated a DA signature (1), or did not detect and/or replicate a DA signature (0). The MRAR of PD to control subjects was used to determine effect direction. Only tested genera that were in common between both datasets were included in the matrix (380 genera for unfiltered data, and 129 for filtered). Then, for each pair of methods in turn, concordances were calculated by summing the number of replicated DA signatures in common between both methods and dividing by the total number of replicated DA signatures. Pairwise concordances, along with the proportion of genera found differentially abundant for each method were visualized for dataset 1, dataset 2, and replicated DA signatures as a boxplot and bar plot respectively using the ggplot2 R package v 3.3.3 [32]. Methods were ordered from lowest (left) to highest (right) overall mean pairwise concordance between dataset 1, dataset 2, and replicated DA signatures. Mean pairwise concordance for each method was calculated in order to see how similar a DA method’s calls were compared to all other methods on average. Methods with a mean pairwise concordance lower than the mean pairwise concordance of all methods in a particular dataset, or for replicated DA signatures, were considered to be part of a “lower concordant” group, while methods with a higher than average mean pairwise concordance were considered to be part of a “higher concordant” group.

### Testing relationship between concordances and proportion of differentially abundant genera

To determine if a relationship existed between concordances and the proportion of genera that were being detected as differentially abundant by each method, both values were plotted against each other for dataset 1, dataset 2, and replicated DA signatures. A trend line was then fitted to the data and added to the plots to visualize any relationship between the two variables. To determine strength and statistical significance of any relationships, a Pearson’s correlation test was performed between concordances and proportion of differentially abundant genera, and results annotated on each plot.

### Effect of taxonomic data filtering on method results and concordances

To determine if taxonomic data filtering has an effect on method results, pairwise concordances, and the relationship between concordances and proportion of differentially abundant genera, pairwise concordances were recalculated for method results derived from performing DA testing on taxonomic data that had rarer genera excluded before analysis. The differences between these concordances and previously derived concordances were calculated to observe how filtering input data affects the resulting concordances between methods. Differences between filtered versus unfiltered data were also calculated for the proportion of differentially abundant genera. Concordances and proportion of differentially abundant genera were then plotted as previously mentioned, with the addition of these differential values. Concordances and proportion of differentially abundant genera were replotted against each other, and retested to observe if any relationships detected with unfiltered data was modified by using filtered data.

### Differentially abundant genera as a function of mean relative abundance and effect size

To determine what type of differentially abundant genera were being detected by methods based on MRA and effect size, the MRAs of tested genera (on log scale) and the log2 fold change of genera in PD were plotted against one another for dataset 1, dataset 2, and replicated signatures. Fisher’s exact test was used to statistically test if detected DA signatures for a particular method were enriched for more, or less, prevalent genera (defined as those that have a MRA above, or below, the median for a dataset or replicated DA signatures). Fisher’s exact test was also used to test if a significant amount of DA signatures for each method were being detected at a particular effect size threshold (absolute fold change in PD of ~ 1.3 or greater), which was chosen based on what was expected to be the smallest meaningful fold change in PD.

### Hierarchical clustering of genera based on similarities in replicated DA signatures

To determine if any groups of DA signatures were being converged upon by all or a subset of methods, hierarchical clustering was performed to group DA signatures based on similarities in DA signature replication between methods. The same binary genus by method matrix used for calculating concordances for replicated DA signatures when performing DA testing on filtered data was used as input to hierarchical clustering, then hierarchical clustering results were visualized in a heatmap using the heatmap.3 function (downloaded from https://raw.githubusercontent.com/obigriffith/biostar-tutorials/master/Heatmaps/heatmap.3.R on 10/21/2019). The default distance function (dist function from stats R package) was used to calculate Euclidean distances between genera and methods. The hierarchical clustering function was specified to be diana from the cluster v 2.1.1 R package. DIANA (DIvisive ANAlysis) performs a divisive hierarchical clustering algorithm [33], which, in this situation, attempts to group DA signatures based on the similarities in replicated DA signatures between methods. The PD to control MRARs and MRAs for each genus were also plotted next to the heatmap. MRARs were given a color gradient from red (lowest MRAR) to light grey (MRAR ~ 1) to blue (highest MRAR). MRAs were given a color gradient from light grey (lowest MRA) to dark green (highest MRA). Hierarchical clustering was also performed for methods in an effort to arrange and group them based on their result similarities.

### Testing for differences between datasets

To test for differences between datasets for various metrics, t-tests were performed and results recorded in the Additional file 1.

## Supplementary Information

**Additional file 1**. Results for t-tests when testing for differences between datasets for various metrics.

**Additional file 2: Table S1**. Method characteristics.

**Additional file 3: Table S2**. List of chosen parameters for each function within each method that either differed from default, or did not have a set default (and therefore a choice had to be made). AIC: Akaike information criterion; FDR: False discovery rate; TMM: Trimmed mean of M-values; RLE: Relative log expression; LDA: Linear discriminant analysis.

**Additional file 4: Table S3**. Dataset 1 false discovery rate (FDR) q-values for differential abundance methods when performed on unfiltered data. Dataset 1 FDR q-values for all differential abundance methods were aggregated into one table along with the mean relative abundance of each genus for PD patients (Case MRA) and control subjects (Control MRA) and the mean relative abundance ratio of Case MRA to Control MRA (MRAR). 201 PD patients and 132 controls were included in all analyses. All genera detected in dataset 1 (445 genera) were included in the analyses. If an analysis resulted in an "NA" for a result, or a result was not outputted by the method, a 1 was placed for the FDR q-value. Calculations for the number of detected DA signatures for each method are located at the bottom of the table. Calculations for the number of methods that detected or replicated a genus as differentially abundant are located to the right of the table.

**Additional file 5: Table S4**. Dataset 2 false discovery rate (FDR) q-values for differential abundance methods when performed on unfiltered data. Dataset 2 FDR q-values for all differential abundance methods were aggregated into one table along with the mean relative abundance of each genus for PD patients (Case MRA) and control subjects (Control MRA) and the mean relative abundance ratio of Case MRA to Control MRA (MRAR). 323 PD patients and 184 controls were included in all analyses. All genera detected in dataset 2 (561 genera) were included in the analyses. If an analysis resulted in an "NA" for a result, or a result was not outputted by the method, a 1 was placed for the FDR q-value. Calculations for the number of detected DA signatures for each method are located at the bottom of the table. Calculations for the number of methods that detected or replicated a genus as differentially abundant are located to the right of the table.

**Additional file 6: Table S5**. Dataset 1 false discovery rate (FDR) q-values for differential abundance methods when performed on filtered data. Dataset 1 FDR q-values for all differential abundance methods were aggregated into one table along with the mean relative abundance of each genus for PD patients (Case MRA) and control subjects (Control MRA) and the mean relative abundance ratio of Case MRA to Control MRA (MRAR). 201 PD patients and 132 controls were included in all analyses. Genera detected in at least 10% of samples (133 genera) were included in the analyses. If an analysis resulted in an "NA" for a result, or a result was not outputted by the method, a 1 was placed for the FDR q-value. Calculations for the number of detected DA signatures for each method are located at the bottom of the table. Calculations for the number of methods that detected or replicated a genus as differentially abundant are located to the right of the table.

**Additional file 7: Table S6**. Dataset 2 false discovery rate (FDR) q-values for differential abundance methods when performed on filtered data. Dataset 2 FDR q-values for all differential abundance methods were aggregated into one table along with the mean relative abundance of each genus for PD patients (Case MRA) and control subjects (Control MRA) and the mean relative abundance ratio of Case MRA to Control MRA (MRAR). 323 PD patients and 184 controls were included in all analyses. Genera detected in at least 10% of samples (195 genera) were included in the analyses. If an analysis resulted in an "NA" for a result, or a result was not outputted by the method, a 1 was placed for the FDR q-value. Calculations for the number of detected DA signatures for each method are located at the bottom of the table. Calculations for the number of methods that detected or replicated a genus as differentially abundant are located to the right of the table.

**Additional file 8: Table S7**. Pairwise concordances between methods with summary statistics calculations. Differential abundance testing was performed using 23 methods on both unfiltered (A-C, 445 genera dataset1, 561 genera dataset 2), and filtered (D-F, 133 genera dataset 1, 195 genera dataset 2) taxonomic data. Values in heatmap cells are pairwise concordances of differential abundance signature calls between two methods. Concordances within each dataset (A,B,D,E) were calculated by first creating a binary taxa by method matrix denoting which methods did (value of 1) or did not (value of 0) detect a certain differential abundance signature, then between each pair of methods, calculating the proportion of detected differential abundance signatures in common out of the total detected between the two methods. Calculation of concordances for replicated differential abundance signatures (C,F) were calculated by first creating a binary taxa by method matrix that denoted whether a signature was replicated (multiple testing corrected significant and same case:control mean relative abundance ratio in both datasets) across datasets (value of 1), or was not replicated (value of 0), then between each pair of methods, calculating the proportion of replicated signatures in common out of the total replicated between the two methods. Cells are colored by a blue (lower concordance) to white (around the mean pairwise concordance of each heatmap) to orange (higher concordance) color gradient. Methods are ordered from lowest (bottom, left) to highest (top, right) mean pairwise concordance. SAMseq did not successfully run for dataset 2 unfiltered data, therefore, table values for SAMseq have been put as "NA" and colored grey in B and C. KW: Kruskal-Wallis; GLM: generalized linear model; CLR: centered log-ratio transformation; TSS: total sum scaling; rCLR: robust CLR with matrix completion using OptSpace algorithm; TMM: trimmed mean of M-values; GLM NBZI: generalized linear model assuming negative binomial distribution with, or without, zero-inflation; RLE: relative log expression.

**Additional file 9: Table S8**. Table further describing heatmap in Fig 4. A table was created to mirror the heatmap in Fig 4 in order to further describe the values being represented in the heatmap visualization and calculate number of methods replicating a particular DA signature. Table rows and columns are ordered to match Fig 4. Values for mean relative abundance ratios and mean relative abundances of each taxon in dataset 1 (MRAR_1 and MRA_1) and dataset 2 (MRAR_2 and MRA_2) from the heatmap in Fig 4 are shown. For each method column, cells are labeled to show what DA signature replicated ("Replicated") or did not replicate ("-") instead of the actual values used for generating the heatmap (1 and 0 respectively). Calculations for the number of methods a DA signature was replicated by are located at the right most column of the table. Only taxa that had at least 1 method replicate it's DA signature are included in the table. Heatmap and hierarchical clustering is based on method results when performed on filtered data (excluding taxa found in < 10% of samples). KW: Kruskal-Wallis; GLM: generalized linear model; CLR: centered log-ratio transformation; TSS: total sum scaling; rCLR: robust CLR with matrix completion using OptSpace algorithm; TMM: trimmed mean of M-values; GLM NBZI: generalized linear model assuming negative binomial distribution with, or without, zero-inflation; RLE: relative log expression.

**Additional file 10: Figure S1**. Detection of differentially abundant genera as a function of mean relative abundance and effect size. To observe what type of differentially abundant genera were being detected by methods based on mean relative abundance and effect size, the mean relative abundances of tested genera (on log scale) and log2 fold change of genera in PD were plotted for dataset 1 (left column), dataset 2 (middle column), and replicated signatures (right column), and repeating these plots for each DA method (rows). Fisher’s exact test was used to determine if DA signatures were enriched for more or less common genera, and genera with absolute fold change of 1.3 or higher. Individual points in the plots represent each tested genus, and is plotted according to that specific genus' mean relative abundance in cases and controls and log2 fold change in PD. For each method, points are shaded black if a method detected a particular genus as differentially abundant, and grey if not detected as such. Horizontal dashed lines represent the median mean relative abundance for either dataset 1, dataset 2, or replicated signatures. Vertical dashed lines represent a fold change of ~1.3 in positive and negative directions. Blue labeled methods are methods who were found to have consistently higher than average concordances in both datasets and replicated signatures, while red labeled methods were found to have consistently lower than average concordances. Grey labeled methods are those that were found to have varied mean concordances. MRA: mean relative abundance, results from Fisher’s exact test testing enrichment of more or less common genera in detected DA signatures; FC: fold change, results from Fisher’s exact test testing enrichment of genera with absolute fold changes > or < 1.3 in detected DA signatures; OR: odds ratio; P: P value of OR; GLM: generalized linear model; CLR: centered log-ratio; KW: Kruskal- Wallis; TSS: total sum scaling (relative abundances); rCLR: robust centered log-ratio transformation with matrix completion; RLE: relative log expression; TMM: trimmed mean of M-values; NBZI: negative binomial zero-inflated.

**Additional file 11: Figure S2**. Detection of differentially abundant genera as a function of mean relative abundance and effect size when performing differential abundance testing on filtered taxonomic data. To observe what type of differentially abundant genera were being detected by methods based on mean relative abundance and effect size when performed on filtered taxonomic data, the mean relative abundances of tested genera (on log scale) and log2 fold change of genera in PD were plotted for dataset 1 (left column), dataset 2 (middle column), and replicated signatures (right column), and repeating these plots for each DA method (rows). Fisher’s exact test was used to determine if DA signatures were enriched for more or less common genera, and genera with absolute fold change of 1.3 or higher. Individual points in the plots represent each tested genus, and is plotted according to that specific genus' mean relative abundance in cases and controls and log2 fold change in PD. Composite mean relative abundances and fold changes were used to plot points for replicated DA signatures by taking the average between datasets. For each method, points are shaded black if a method detected a particular genus as differentially abundant, and grey if not detected as such. Horizontal dashed lines represent the median mean relative abundance for either dataset 1, dataset 2, or replicated signatures. Vertical dashed lines represent a fold change of ~1.3 in positive and negative directions. Blue labeled methods are methods who were found to have consistently higher than average concordances in both datasets and replicated signatures, while red labeled methods were found to have consistently lower than average concordances. Grey labeled methods are those that were found to have varied mean concordances. MRA: mean relative abundance, results from Fisher’s exact test testing enrichment of more or less common genera in detected DA signatures; FC: fold change, results from Fisher’s exact test testing enrichment of genera with absolute fold changes > or < 1.3 in detected DA signatures; OR: odds ratio; P: P value of OR; GLM: generalized linear model; CLR: centered log-ratio; KW: Kruskal- Wallis; TSS: total sum scaling (relative abundances); rCLR: robust centered log-ratio transformation with matrix completion; RLE: relative log expression; TMM: trimmed mean of M-values; NBZI: negative binomial zero-inflated.

## Data Availability

Individual-level raw sequences and basic metadata of both datasets analyzed in the current manuscript are publicly available at NCBI Sequence Read Archive (SRA) under BioProject PRJNA601994. Source code and input phyloseq objects used to perform analyses in the current manuscript are publicly available within a Github repository, which can be accessed using the following link: https://github.com/zwallen/Wallen_DAMethodCompare_2021. FDR q-values for each differential abundance method can be found in Additional files 4, 5, 6, 7: Tables S3–S6.

## Abbreviations

DA: Differential abundance
PD: Parkinson disease
GLM: Generalized linear model
FPR: False positive rate
FDR: False discovery rate
TSS: Total sum scaling
BH: Benjamini-Hochberg
NBZI: Negative binomial zero-inflated
CLR: Centered log-ratio
rCLR: Robust centered log-ratio
CSS: Cumulative sum scaling
TMM: Trimmed mean of M-values
RLE: Relative log expression
MRAR: Mean relative abundance ratio
MRA: Mean relative abundance
OR: Odds ratio
