# Supplementary material for: Comparison study of differential abundance testing methods using two large Parkinson disease gut microbiome datasets derived from 16S amplicon sequencing
Source: BMC Bioinformatics. 2021 May 25;22:265. doi: 10.1186/s12859-021-04193-6 (PMC8147401; doi:10.1186/s12859-021-04193-6)
Supplement: Supplementary file 10 — Additional file 10: Figure S1. Detection of differentially abundant genera as a function of mean relative abundance and effect size. To observe what type of differentially abundant genera were being detected by methods based on mean relative abundance and effect size, the mean relative abundances of tested genera (on log scale) and log2 fold change of genera in PD were plotted for dataset 1 (left column), dataset 2 (middle column), and replicated signatures (right column), and repeating these plots for each DA method (rows). Fisher’s exact test was used to determine if DA signatures were enriched for more or less common genera, and genera with absolute fold change of 1.3 or higher. Individual points in the plots represent each tested genus, and is plotted according to that specific genus' mean relative abundance in cases and controls and log2 fold change in PD. For each method, points are shaded black if a method detected a particular genus as differentially abundant, and grey if not detected as such. Horizontal dashed lines represent the median mean relative abundance for either dataset 1, dataset 2, or replicated signatures. Vertical dashed lines represent a fold change of ~1.3 in positive and negative directions. Blue labeled methods are methods who were found to have consistently higher than average concordances in both datasets and replicated signatures, while red labeled methods were found to have consistently lower than average concordances. Grey labeled methods are those that were found to have varied mean concordances. MRA: mean relative abundance, results from Fisher’s exact test testing enrichment of more or less common genera in detected DA signatures; FC: fold change, results from Fisher’s exact test testing enrichment of genera with absolute fold changes > or < 1.3 in detected DA signatures; OR: odds ratio; P: P value of OR; GLM: generalized linear model; CLR: centered log-ratio; KW: Kruskal- Wallis; TSS: total sum scaling (relative abundances); rCLR: [file 12859_2021_4193_MOESM10_ESM.pdf]

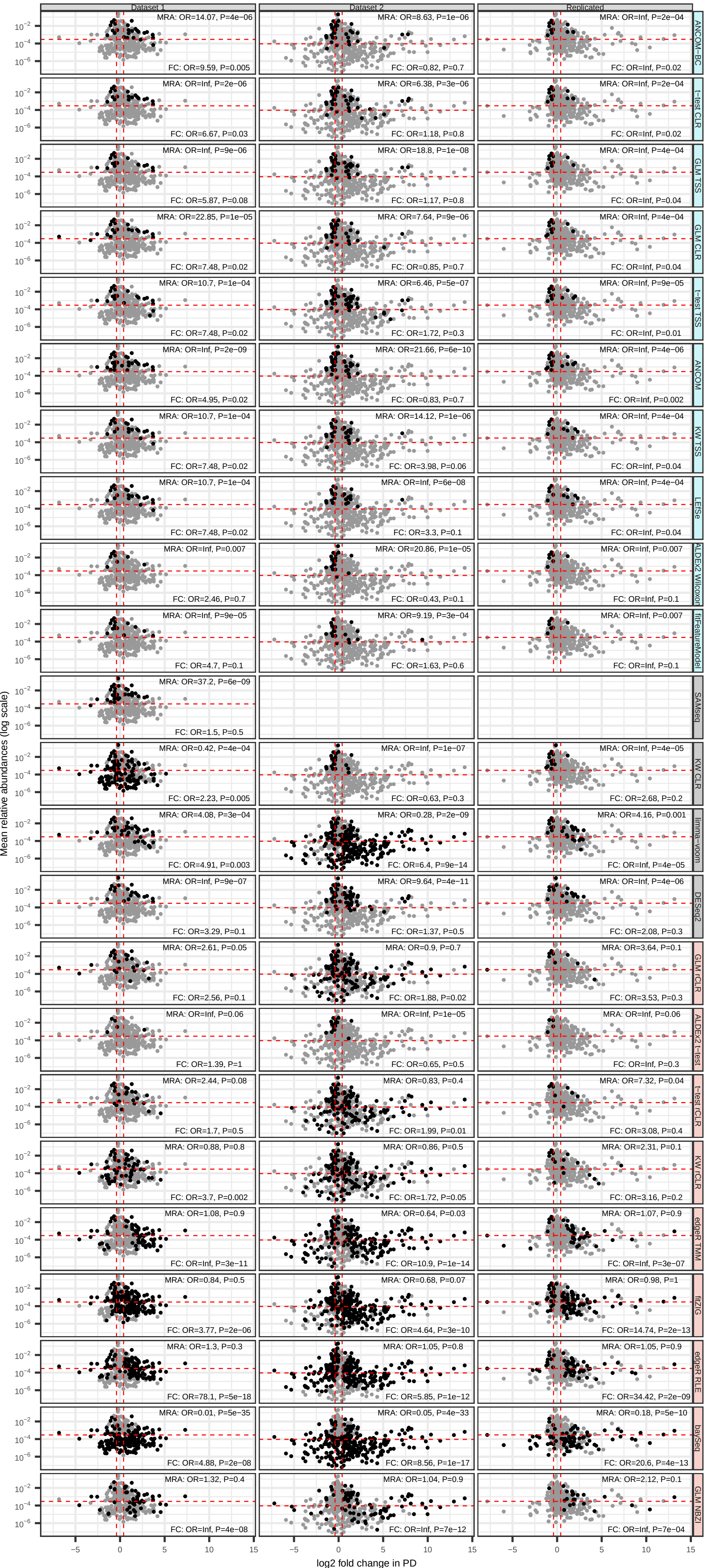
Dataset 1
Dataset 2
Replicated
ANCOM−BC
MRA: OR=14.07, P=4e−06
FC: OR=9.59, P=0.005
MRA: OR=8.63, P=1e−06
FC: OR=0.82, P=0.7
MRA: OR=Inf, P=2e−04
FC: OR=Inf, P=0.02
t−test CLR
MRA: OR=Inf, P=2e−06
FC: OR=6.67, P=0.03
MRA: OR=6.38, P=3e−06
FC: OR=1.18, P=0.8
MRA: OR=Inf, P=2e−04
FC: OR=Inf, P=0.02
GLM TSS
MRA: OR=Inf, P=9e−06
FC: OR=5.87, P=0.08
MRA: OR=18.8, P=1e−08
FC: OR=1.17, P=0.8
MRA: OR=Inf, P=4e−04
FC: OR=Inf, P=0.04
GLM CLR
MRA: OR=22.85, P=1e−05
FC: OR=7.48, P=0.02
MRA: OR=7.64, P=9e−06
FC: OR=0.85, P=0.7
MRA: OR=Inf, P=4e−04
FC: OR=Inf, P=0.04
t−test TSS
MRA: OR=10.7, P=1e−04
FC: OR=7.48, P=0.02
MRA: OR=6.46, P=5e−07
FC: OR=1.72, P=0.3
MRA: OR=Inf, P=9e−05
FC: OR=Inf, P=0.01
ANCOM
MRA: OR=Inf, P=2e−09
FC: OR=4.95, P=0.02
MRA: OR=21.66, P=6e−10
FC: OR=0.83, P=0.7
MRA: OR=Inf, P=4e−06
FC: OR=Inf, P=0.002
KW TSS
MRA: OR=10.7, P=1e−04
FC: OR=7.48, P=0.02
MRA: OR=14.12, P=1e−06
FC: OR=3.98, P=0.06
MRA: OR=Inf, P=4e−04
FC: OR=Inf, P=0.04
LEfSe
MRA: OR=10.7, P=1e−04
FC: OR=7.48, P=0.02
MRA: OR=Inf, P=6e−08
FC: OR=3.3, P=0.1
MRA: OR=Inf, P=4e−04
FC: OR=Inf, P=0.04
ALDEx2 Wilcoxon
MRA: OR=Inf, P=0.007
FC: OR=2.46, P=0.7
MRA: OR=20.86, P=1e−05
FC: OR=0.43, P=0.1
MRA: OR=Inf, P=0.007
FC: OR=Inf, P=0.1
fitFeatureModel
MRA: OR=Inf, P=9e−05
FC: OR=4.7, P=0.1
MRA: OR=9.19, P=3e−04
FC: OR=1.63, P=0.6
MRA: OR=Inf, P=0.007
FC: OR=Inf, P=0.1
SAMseq
MRA: OR=37.2, P=6e−09
FC: OR=1.5, P=0.5
KW CLR
MRA: OR=0.42, P=4e−04
FC: OR=2.23, P=0.005
MRA: OR=Inf, P=1e−07
FC: OR=0.63, P=0.3
MRA: OR=Inf, P=4e−05
FC: OR=2.68, P=0.2
limma−voom
MRA: OR=4.08, P=3e−04
FC: OR=4.91, P=0.003
MRA: OR=0.28, P=2e−09
FC: OR=6.4, P=9e−14
MRA: OR=4.16, P=0.001
FC: OR=Inf, P=4e−05
DESeq2
MRA: OR=Inf, P=9e−07
FC: OR=3.29, P=0.1
MRA: OR=9.64, P=4e−11
FC: OR=1.37, P=0.5
MRA: OR=Inf, P=4e−06
FC: OR=2.08, P=0.3
GLM rCLR
MRA: OR=2.61, P=0.05
FC: OR=2.56, P=0.1
MRA: OR=0.9, P=0.7
FC: OR=1.88, P=0.02
MRA: OR=3.64, P=0.1
FC: OR=3.53, P=0.3
ALDEx2 t−test
MRA: OR=Inf, P=0.06
FC: OR=1.39, P=1
MRA: OR=Inf, P=1e−05
FC: OR=0.65, P=0.5
MRA: OR=Inf, P=0.06
FC: OR=Inf, P=0.3
t−test rCLR
MRA: OR=2.44, P=0.08
FC: OR=1.7, P=0.5
MRA: OR=0.83, P=0.4
FC: OR=1.99, P=0.01
MRA: OR=7.32, P=0.04
FC: OR=3.08, P=0.4
KW rCLR
MRA: OR=0.88, P=0.8
FC: OR=3.7, P=0.002
MRA: OR=0.86, P=0.5
FC: OR=1.72, P=0.05
MRA: OR=2.31, P=0.1
FC: OR=3.16, P=0.2
edgeR TMM
MRA: OR=1.08, P=0.9
FC: OR=Inf, P=3e−11
MRA: OR=0.64, P=0.03
FC: OR=10.9, P=1e−14
MRA: OR=1.07, P=0.9
FC: OR=Inf, P=3e−07
fitZIG
MRA: OR=0.84, P=0.5
FC: OR=3.77, P=2e−06
MRA: OR=0.68, P=0.07
FC: OR=4.64, P=3e−10
MRA: OR=0.98, P=1
FC: OR=14.74, P=2e−13
edgeR RLE
MRA: OR=1.3, P=0.3
FC: OR=78.1, P=5e−18
MRA: OR=1.05, P=0.8
FC: OR=5.85, P=1e−12
MRA: OR=1.05, P=0.9
FC: OR=34.42, P=2e−09
baySeq
MRA: OR=0.01, P=5e−35
FC: OR=4.88, P=2e−08
MRA: OR=0.05, P=4e−33
FC: OR=8.56, P=1e−17
MRA: OR=0.18, P=5e−10
FC: OR=20.6, P=4e−13
GLM NBZI
MRA: OR=1.32, P=0.4
FC: OR=Inf, P=4e−08
MRA: OR=1.04, P=0.9
FC: OR=Inf, P=7e−12
MRA: OR=2.12, P=0.1
FC: OR=Inf, P=7e−04
Mean relative abundances (log scale)
log2 fold change in PD

Fig. S1. Detection of differentially abundant genera as a function of mean relative abundance and effect size.

To observe what type of differentially abundant genera were being detected by methods based on mean relative abundance and effect size, the mean relative abundances of tested genera (on log scale) and log2 fold change of genera in PD were plotted for dataset 1 (left column), dataset 2 (middle column), and replicated signatures (right column), and repeating these plots for each DA method (rows). Fisher's exact test was used to determine if DA signatures were enriched for more or less common genera, and genera with absolute fold change of 1.3 or higher. Individual points in the plots represent each tested genus, and is plotted according to that specific genus' mean relative abundance in cases and controls and log2 fold change in PD. Composite mean relative abundances and fold changes were used to plot points for replicated DA signatures by taking the average between datasets. For each method, points are shaded black if a method detected a particular genus as differentially abundant, and grey if not detected as such. Horizontal dashed lines represent the median mean relative abundance for either dataset 1, dataset 2, or replicated signatures. Vertical dashed lines represent a fold change of ~1.3 in positive and negative directions. Blue labeled methods are methods who were found to have consistently higher than average concordances in both datasets and replicated signatures, while red labeled methods were found to have consistently lower than average concordances. Grey labeled methods are those that were found to have varied mean concordances. MRA: mean relative abundance, results from Fisher's exact test testing enrichment of more or less common genera in detected DA signatures; FC: fold change, results from Fisher's exact test testing enrichment of genera with absolute fold changes > or < 1.3 in detected DA signatures; OR: odds ratio; P: *P* value of OR; GLM: generalized linear model; CLR: centered log-ratio; KW: Kruskal-Wallis; TSS: total sum scaling (relative abundances); rCLR: robust centered log-ratio transformation with matrix completion; RLE: relative log expression; TMM: trimmed mean of M-values; NBZI: negative binomial zero-inflated.
